# Metronidazole versus lactic acid for treating bacterial vaginosis (VITA): protocol for a randomised controlled trial to assess the clinical and cost effectiveness of topical lactic acid gel for treating second and subsequent episodes of bacterial vaginosis

**DOI:** 10.1186/s13063-019-3731-7

**Published:** 2019-11-27

**Authors:** Lindsay Armstrong-Buisseret, Clare Brittain, Miruna David, Gillian Dean, Frances Griffiths, Trish Hepburn, Louise Jackson, Joe Kai, Alan Montgomery, Tracy Roberts, Sukhwinder Thandi, Jonathan D. C. Ross

**Affiliations:** 1 Nottingham Clinical Trials Unit (NCTU), Building 42, University of Nottingham, University Park, Nottingham, NG7 2RD UK; 2 Clinical Microbiology, University Hospitals Birmingham NHS Foundation Trust, Queen Elizabeth Hospital Birmingham, Mindelsohn Way, Edgbaston, Birmingham, B15 2GW UK; 3 Elton John Research Centre, Sussex House, 1 Abbey Road, Brighton, BN2 1ES UK; 4 Warwick Medical School, University of Warwick, Coventry, CV4 7AL UK; 5 Health Economics Unit, Institute of Applied Health Research, College of Medical and Dental Sciences, University of Birmingham, Edgbaston, Birmingham, B15 2TT UK; 6 School of Medicine, Tower Building, University of Nottingham, University Park, Nottingham, NG7 2RD UK; 7 Department of GU Medicine, University Hospitals Birmingham NHS Foundation Trust, Whittall Street Clinic, Whittall Street, Birmingham, B4 6DH UK

**Keywords:** Bacterial vaginosis, Lactic acid gel, Metronidazole, Recurrence, VITA, Antibiotic usage, Cost-benefit analysis

## Abstract

**Background:**

Bacterial vaginosis (BV) affects 30–50% of women at some time in their lives and is an embarrassing and distressing condition which can be associated with potentially serious comorbidities. Current antibiotic treatments such as metronidazole are effective but can result in side effects, and recurrence is common. This trial aims to investigate whether lactic acid gel is clinically effective and cost effective in the treatment of recurrent BV compared with metronidazole.

**Methods:**

VITA is an open-label, multicentre, parallel group randomised controlled trial for women with a clinical diagnosis of BV and at least one previous BV episode in the past 2 years. Participants will be randomised 1:1 to intravaginal lactic acid gel 5 ml once daily for 7 days or oral metronidazole tablets 400 mg twice daily for 7 days. All participants will be followed up for 6 months to assess health status and healthcare costs. A subgroup will be interviewed to further explore adherence, tolerability and acceptability of treatment. The estimated sample size is 1900 participants to detect a 6% absolute increase in response rate to 86% in those receiving lactic acid gel. The primary outcome is participant-reported resolution of BV at Week 2.

**Discussion:**

Results from this trial will help inform UK treatment guidelines for BV and may provide an alternative effective treatment for recurrent episodes of this condition which avoids repeated exposure to antibiotics.

**Trial registration:**

ISRCTN, ISRCTN14161293. Registered on 8 September 2017.

## Background

Bacterial vaginosis (BV) is a common condition which causes an offensive smelling vaginal discharge and is associated with an increased risk of HIV acquisition and transmission, pelvic inflammatory disease and adverse pregnancy outcomes [1–4]. The normal bacteria found in the vagina include numerous lactobacilli which produce lactic acid maintaining a low pH and inhibiting the growth of other bacteria. In BV the pH rises in association with loss of lactobacilli and there is an overgrowth of anaerobic bacteria.

Current treatment with oral antibiotics to reduce the associated overgrowth of bacteria in the vagina can be effective in the short term, but it is frequently associated with side effects and 30% rate of recurrence over the subsequent 3 months [5–8], necessitating repeated antibiotic treatment. Knowledge of the underlying pathogenesis of BV is limited and the factor(s) initiating BV are unknown, although viral bacteriophages, novel sexually transmitted bacteria, bacterial biofilms and disruption to the bacterial microbiome have all been proposed [9].

BV was diagnosed in 100,636 women attending sexual health clinics in England in 2014, and around 30,000 women will have recurrent BV within 3 months of their initial treatment. The prevalence of BV has not changed significantly over the past 5 years (https://www.gov.uk/government/statistics/sexually-transmitted-infections-stis-annual-data-tables).

The recurrent nature of BV leads to frequent use of antibiotics. The use of lactic acid gel as treatment would reduce antibiotic exposure in the population as recommended in the ‘Action plan to support the UK antimicrobial resistance strategy 2013 to 2018’ (www.gov.uk/government/publications/uk-5-year-antimicrobial-resistance-strategy-2103-to-2018) and the ‘European strategic action plan on antibiotic resistance 2011–2016’ (www.euro.who.int/_data/assets/pdf_file/0011/148988/RC61_Pres_Rodier_antibiotic_resistance.pdf).

Lactic acid gel (pH 4.5) used intravaginally replicates the production of lactic acid by lactobacilli in the normal vagina. Previous small studies of daily intravaginal acid gel or pessary for the treatment of BV have reported inconsistent results, with efficacy ranging between 18 and 100% [6, 10–15]. The regimen used most commonly in previous trials was once daily application for 1 week, and an increased frequency of dosing did not affect the response rate (efficacy 23–93% for once daily versus 18–100% for twice daily). To permit comparison with previous trials, to maximise participant acceptability and because the effectiveness of this regimen remains unconfirmed, we propose using 4.5% lactic acid gel inserted intravaginally once a day for 7 days. Although the use of topical lactic acid gel is not recommended in current BV treatment guidelines due to a lack of evidence from well-designed randomised trials [7], the proposed trial will advance our understanding by assessing whether intravaginal lactic acid gel is effective and well tolerated for the treatment of recurrent BV, and can reduce antibiotic usage in this large group of women.

The factors affecting the acceptability of topical treatment for BV are not known, and a qualitative assessment of adherence to and acceptability of treatment, and how these can be improved, will be performed.

BV is a common disease with serious physical and psychological sequelae. There is therefore the potential for substantial health gain if a more effective and well-tolerated regimen can be identified, which also reduces antibiotic exposure. The prospects for the study findings to influence clinical practice are high based on the multicentre approach including primary care, robust study design, existing availability of lactic acid gel and identified need to limit antibiotic use to reduce the development of antimicrobial resistance.

### Justification for design

This trial will compare the effectiveness, tolerability, adherence, acceptability and cost effectiveness in participants with BV randomised to intravaginal lactic acid gel (intervention) versus those randomised to oral metronidazole (control). A pragmatic trial design is used to maximise its relevance to patients and clinicians and to facilitate rapid adoption into clinical practice. A qualitative assessment will also investigate the acceptability of treatment, and if necessary, address issues raised by participants.

A superiority design has been chosen and powered to detect an absolute difference of 6% in the resolution of BV between the two treatment arms, with an assumed effectiveness of oral metronidazole of 80% [16–19].

There will be no blinding of participants to treatment, as this will maximise treatment compliance and allow comparison of the acceptability of the two treatments. A double placebo (“double dummy”) design for oral versus vaginal modes of treatment delivery was not considered to be acceptable to most women.

Participants will take their own vaginal samples at baseline and at the Week 2 follow-up. The taking of such samples is widely used in clinical practice and is acceptable to women. At their baseline visit, participants will be instructed on how to take their own vaginal samples; they will then take their own baseline samples, which will be shipped to a central laboratory by site staff. Before they leave the clinic (or General Practitioner [GP] practice), participants will be provided with a second sampling kit and instructions for taking their own vaginal samples at home at Week 2.

After the baseline visit, participants will not be required to attend any further face-to-face visits, reflecting usual clinical practice. All follow-up data will be collected by participants via online questionnaires.

### Choice of treatment

The aim of this trial is to determine if using lactic acid gel in the vagina to ‘replace’ vaginal acidity is better than oral metronidazole for treatment of BV. Some previous studies have suggested that this approach could be successful, but they are not conclusive, and current guidelines highlight a need for more evidence before recommending the use of intravaginal lactic acid gel.

Previous small studies of daily intravaginal acid gel or pessary for the treatment of BV have reported inconsistent results with efficacies of 18–100% [6, 10–15, 20]. The regimen used most commonly in previous trials was once daily application for 7 days, and an increased frequency of dosing did not affect the response rate (efficacy 23–93% for once/day compared with 18–100% for twice/day). To permit comparison with previous trials, the intervention group will receive a regimen of lactic acid gel inserted intravaginally once a day for 7 days, to maximise participant acceptability and because the effectiveness of this regimen remains unconfirmed.

If effective, the use of lactic acid gel will result in decreased antibiotic treatment use, which will maintain the balance between the gut bacteria (microbiome) for individual participants and reduce the potential for development of antimicrobial resistance in the community. In addition, it will provide an alternative treatment for women who have failed to respond to current treatment for BV with systemic antibiotics.

The control group will receive a 7-day course of twice daily 400 mg oral metronidazole. This has been chosen as the comparator because it is recommended as first line therapy in the UK national BV treatment guideline [7], it is active against a wide range of the anaerobic bacteria associated with BV and it is commonly used in clinical practice supported by evidence from randomised controlled trials [18].

## Methods/design

### Aims and objectives

The trial hypothesis is that intravaginal lactic acid gel is clinically effective and cost effective in the treatment (i.e. resolution of symptoms) of women with recurrent BV compared with oral metronidazole. The aim is to test this hypothesis by comparing symptom resolution in the intervention group (receiving intravaginal lactic acid gel) with the control group (receiving oral metronidazole) for women with recurrent BV.

The primary objective of the trial is to determine whether intravaginal lactic acid gel is better than oral metronidazole for symptomatic resolution of recurrent BV. Secondary objectives include the following: comparing the time to first recurrence of BV symptoms; comparing the frequency of BV episodes over 6 months; comparing the frequency of BV treatments required over 6 months; comparing microbiological resolution of BV on microscopy 2 weeks after presentation; comparing the tolerability profiles of lactic acid gel and metronidazole; comparing adherence to lactic acid gel versus metronidazole tablets; comparing acceptability of use of lactic acid gel versus metronidazole tablets; determining comparative presence of concurrent sexually transmitted infections (STIs) at baseline and at Week 2; comparing quality of life (measured using the SF-12™ Health Survey [21]); comparing the cost effectiveness of using intravaginal lactic acid gel versus oral metronidazole tablets. In addition, samples for further microbiological analysis, including gene sequencing, will be collected for future investigation into the factors associated with successful treatment.

### Outcome measures

The primary outcome is resolution of BV based on participant-reported resolution of symptoms at Week 2. Secondary outcome measures are as follows: time to first recurrence of BV; number of participant-reported BV episodes over 6 months; number of participant-reported BV treatment courses over 6 months; microbiological resolution of BV on microscopy of vaginal smears at Week 2; comparative tolerability of lactic acid gel and metronidazole assessed by web-based participant reporting of side effects (including nausea, vomiting, taste disturbance, vaginal irritation, diarrhoea and abdominal pain) and via participant telephone interviews; participant-reported adherence to treatment; acceptability of treatments via qualitative assessment in a subgroup of participants; prevalence of concurrent STIs (gonorrhoea, chlamydia and trichomoniasis) at baseline and at Week 2; quality of life as assessed by SF-12™ Health Survey at baseline, Week 2 and 6 months; comparative cost effectiveness of using intravaginal lactic acid gel versus oral metronidazole tablets via National Health Service (NHS) Service use questionnaire.

### Design and setting

This is an open-label, multicentre, parallel group, randomised controlled trial. Participants will be randomised 1:1 to receive either intravaginal lactic acid gel treatment or oral metronidazole tablets.

**Fig. 1:**
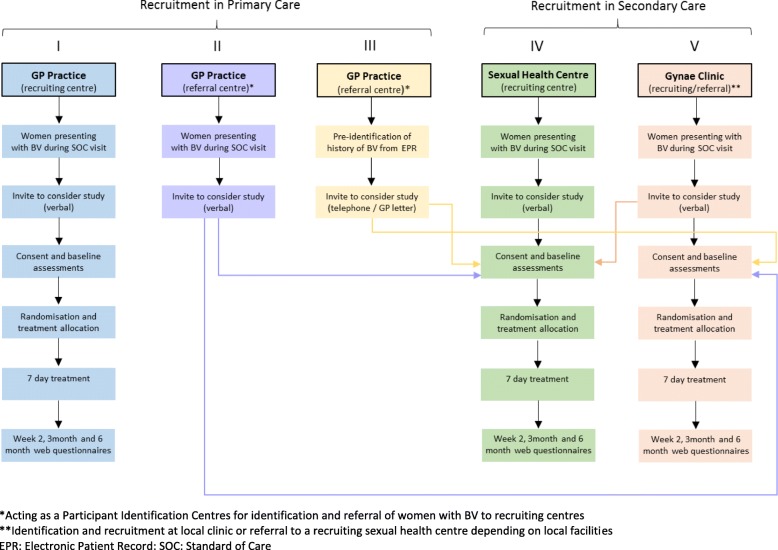
VITA participant pathways in primary and secondary care settings

Women will be recruited from primary care (GP) practices and sexual health outpatient and gynaecology clinics in the UK (Fig. 1).

The Standard Protocol Items: Recommendations for Interventional Trials (SPIRIT) checklist is provided as Additional file 1.

#### Primary care (GP practices)

Primary care practices will involve the following:

1. Opportunistic identification of women presenting with BV in GP practices which are VITA recruiting centres with trained research staff on-site. Participants will be identified, consented, randomised and prescribed trial treatment at the practice. These research-ready sites require on-site availability of trained research nurses and facilities to directly consent and randomise patients.
2. Opportunistic identification and referral of women with BV attending GP practices without on-site research staff, i.e. Participant Identification Centres, to refer women presenting with BV to local participating VITA recruiting centres for invitation to participate in the trial.
3. Pre-identification of women with a history of BV by GPs from electronic patient records/primary care databases. GPs will provide potential participants with information on the trial via telephone or letter and invite them to attend a local recruiting centre for consent if they develop BV and are interested in participating.

#### Secondary care

Secondary care practices will involve the following:

1. Opportunistic identification of women presenting with BV in sexual health centres which are VITA recruiting centres with trained research staff on-site. Participants will be identified, consented, randomised and dispensed trial treatment at the centre. These research-ready sites require on-site availability of trained research nurses and facilities to directly consent and randomise patients.
2. Opportunistic identification of women presenting with BV in gynaecology clinics which either (a) are VITA recruiting centres with trained research staff on-site where participants would be identified, consented, randomised and prescribed trial treatment within the clinic, or (b) act as VITA referral clinics (Participant Identification Centres) where women presenting with BV may be referred to a nearby participating recruiting sexual health centre for invitation to participate in the trial.

### Participants

**Fig. 2:**
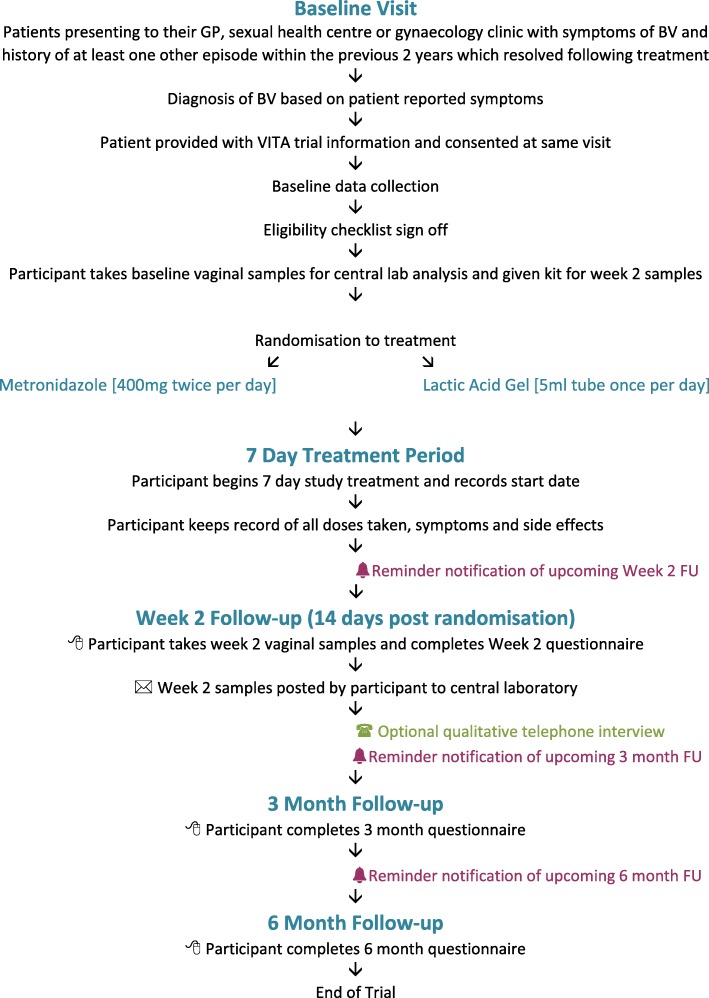
Participant flow

The flow of participants from presentation through to follow-up is shown in Fig. 2. Inclusion criteria are as follows: age 16 years or over; clinical diagnosis of BV based on patient-reported symptoms of discharge with an unpleasant (typically fishy) odour (with or without positive microscopy according to local site practice); history of at least one previous episode of BV within the past 2 years (clinically diagnosed or patient reported) which resolved with treatment; willing to use either intravaginal lactic acid gel or oral tablets for the management of BV; willing to take own vaginal samples; willing to avoid vaginal douching during treatment; willing to provide contact details and be contacted for the purpose of collecting follow-up information; willing to avoid sexual intercourse or use effective contraception for the 7-day duration of study treatment (condoms are not considered to be effective contraception due to a potential interaction with lactic acid gel); access to the Internet and email and willing to complete web-based follow-up questionnaires in English; provision of written informed consent.

Exclusion criteria are as follows: contraindications or allergy to lactic acid gel or metronidazole tablets; pregnant or breastfeeding; patients currently trying to conceive; use of oral antibiotics (other than the study treatment) or antifungal agents concurrently, within the last 2 weeks or planned use within the next 2 weeks; use of topical vaginal antibiotics, antifungals or acidifying products (other than the study treatment) concurrently, within the last 2 weeks, or planned use within the next 2 weeks; previous participation in this study; current participation in another trial involving an investigational medicinal product (IMP).

### Contraindications and concomitant medications

#### Metronidazole

As per exclusion criteria, any known hypersensitivity to metronidazole, other nitroimidazole derivatives or any of the ingredients in metronidazole tablets will exclude patients from the trial. The metronidazole summary of product characteristics (SmPC) gives further details, but the following should be noted:

- Alcohol should be avoided (including products containing alcohol) during the course of treatment and for 48 h afterwards.
- Elevated international normalised ratio (INR) and bleeding events have been reported with concurrent use of warfarin and metronidazole.

#### Lactic acid gel

There is no SmPC for lactic acid gel, but the following should be noted:

- Shellfish allergy: Some lactic acid gel brands may contain glycogen obtained from oysters.
- Condom use: The effects of lactic acid gel on condom degradation have not been fully determined. Therefore, it is advised that condoms should not be assumed to be an effective method of contraception during the 7-day treatment period with lactic acid gel.

#### Concomitant medications

Concomitant medications relevant to BV, such as oral or topical antibiotics and/or antifungals, should be recorded at baseline to determine patient eligibility. The use of additional treatments is permitted after completion of the 7-day study treatment at the discretion of the participant’s physician.

### Screening and consent

Women either pre-identified by, or presenting to, referring or recruiting GP practices, sexual health centres or gynaecology clinics with symptoms of BV will be approached by a member of the site research team to determine whether they are interested in participating in the trial. They will be given a verbal explanation of the trial along with a Participant Information Sheet, and they will have time to read this and ask any questions about the trial prior to consent. Written informed consent will be requested during the same clinic visit.

Separate optional consent will be required for any participants who are interested in taking part in the qualitative telephone interview. Participants recruited from sexual health centres and gynaecology clinics will additionally be asked for their optional consent to inform their GP that they are taking part in the trial.

### Randomisation

Randomisation will take place in primary care (GP) practices or outpatient sexual health/gynaecology clinics. Participants will be randomised 1:1 to lactic acid gel or metronidazole using a remote Internet-based randomisation system developed and maintained by the Nottingham Clinical Trials Unit (NCTU). The concealed allocation system will use a minimisation algorithm with the following variables and levels: site, type of site (GP practice, sexual health clinic, gynaecology clinic), number of episodes of BV in the previous 12 months (0, 1–3, > 3) and whether the participant has had a female sexual partner in the previous 12 months (yes/no). The allocation system will be created by the NCTU in accordance with their standard operating procedure (SOP) and held on a secure University of Nottingham server.

As this is an open-label trial, there will be no blinding of the participants, investigator, site research team or trial team to treatment allocation. However, the central laboratory staff performing BV microscopy and STI testing will be blinded to the participants’ treatment allocation, and all analyses that present data separately by treatment arm or that estimate between-group effects will be conducted by a statistician blinded to treatment allocation.

### Trial intervention

There are two treatment arms within the trial:

- Lactic acid gel; 5 ml to be inserted into the vagina before bedtime once per day for 7 days
- Metronidazole tablets; 400 mg to be taken orally twice daily, approximately 12 h apart, for 7 days. Tablets should be swallowed whole, taken during or after meals with a glass of water and not crushed or chewed.

#### Investigational medicinal product

Metronidazole tablets are an IMP in the trial and are licensed for use in the treatment of BV as per the SmPC.

#### Medical device

Lactic acid gel is a registered medical device consisting of a colourless viscous gel administered through an intravaginal tube applicator. Known side effects of lactic acid gel include vaginal irritation, e.g. redness, stinging and itching. In rare cases an allergic skin reaction, e.g. severe redness, swelling or burning, may occur.

### Treatment supplies, labelling and storage

Participants will receive their study treatment via the routine method of dispensing used in the setting of the recruiting centre. In sexual health centres and gynaecology clinics, this may be via issuance from standard clinic stocks. In primary (GP) care practices, allocated treatments may be issued via standard prescription as per routine care. Participants will be advised to obtain their allocated study treatment from any dispensing pharmacy. Any licensed brands of metronidazole or lactic acid gel may be used.

Trial-specific labelling will not be required, as the IMP is being used within the terms of its marketing authorisation in the UK. The IMP will be dispensed to a participant in accordance with a prescription given by an authorised healthcare professional and labelled in accordance with the requirements of Schedule 5 to the Medicines for Human Use (SI 1994/31 94) (Marketing Authorisations Etc.) Regulations 1994 that apply in relation to relevant dispensed medicinal products.

The IMP should be stored as per manufacturer’s instructions. Recruiting sexual health centres and gynaecology clinics should record batch number(s) and manufacturer(s) of all allocated treatments.

### Dosing schedule

Treatment should be started on the day of receipt, but participants will be instructed to record their actual start date and time (morning or evening) of dosing in their patient diaries. Participants will also be asked to use their patient diaries to log all subsequent doses taken and/or missed doses over the treatment period in order to aid compliance to the treatment schedule.

No treatment or dose modifications are expected in this trial. Where a dose is accidentally missed, participants will be advised to follow the manufacturer’s instructions or seek advice from their physician. In the case of any missed dose, participants will be advised to continue to complete their treatment course.

### Trial assessments and procedures

**Fig. 3:**
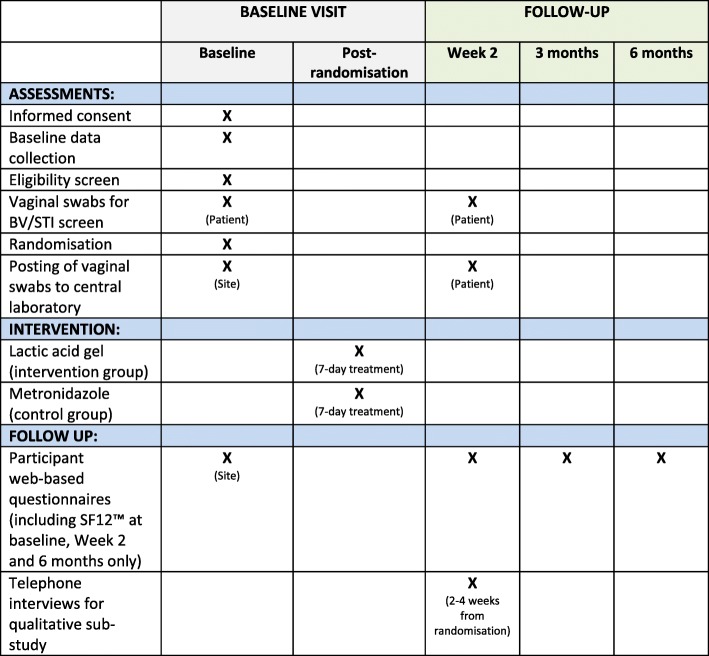
Summary of assessments at baseline and follow-up

All assessments and procedures to be performed at each time point for participants are indicated in Fig. 3. Most assessments will be done at baseline, including demographics, symptoms and previous BV episodes, medical and sexual history, concomitant medications, contraception and condom use, SF12™ Health Survey and vaginal samples for BV/STI screening (sites will send the baseline samples to a central laboratory at University Hospitals Birmingham NHS Foundation Trust which is accredited under the UK Accreditation Service to perform the tests). After randomisation into the trial, participants will then take their first dose of trial treatment and continue taking trial treatment for 7 days. At Week 2, participants will take their own vaginal samples and send them to the central laboratory. They will also complete a web-based questionnaire with details of symptoms, treatment adherence and tolerability, any known side effects, healthcare use, additional BV treatments, sexual history, contraception/condom use and another SF12™ Health Survey. Participants will be asked to complete a further two web-based questionnaires at 3 months and 6 months with details of BV recurrence, sexual history, healthcare use, additional BV treatments, contraception/condom use and a final SF12™ Health Survey (6 months only). Those not responding to requests to complete web-based questionnaires will be contacted by phone and/or text to collect follow-up data.

Participants can discontinue trial treatment at any time but can remain in the trial, taking Week 2 vaginal samples and completing all follow-up questionnaires. They can also withdraw from the follow-up assessments at any time.

### Qualitative telephone interviews

A subgroup of participants will be contacted for semi-structured telephone interviews to explore acceptability and adherence to trial treatment, and how these can be optimised. A random sample of participants who gave optional consent to be contacted will be recruited until initial analysis suggests data saturation. The sample size will then be reviewed to check that it is representative of the trial target population in terms of age, ethnicity, recruiting site and number of BV episodes. Where necessary, further participants will be recruited to ensure a diverse spread of the target population. The final sample size is expected to be approximately 30 participants (15 in each trial arm). Interviews will be audio recorded, transcribed and uploaded into NVivo to assist with data management. Transcripts will be coded and analysed thematically.

### Collection and analysis of vaginal samples

At the baseline visit, participants will be provided with a kit for taking their own vaginal samples at Week 2. The central laboratory will perform the following tests on vaginal samples taken at baseline and at Week 2: microscopic assessment of BV; nucleic acid amplification tests for chlamydia, gonorrhoea and trichomoniasis. Results will be returned within 1–2 months for the recruiting site to review (investigator and research nurse) and to arrange further testing or treatment according to local protocols. These trial tests will not form the basis for patient management at the baseline visit; clinicians will take additional tests processed locally to inform immediate patient care as indicated by the patient’s clinical presentation.

### Adverse events and pregnancy reporting

The safety profiles of the treatments in this trial are well characterised. Metronidazole is being used for its licensed indication and lactic acid gel is being used within its intended use covered by the CE mark. In order to provide secondary outcome data to compare tolerability of the two treatments, specified adverse reactions experienced during trial treatment will be reported. The following are regarded as expected for the purpose of this trial and will be reported on the Week 2 questionnaire completed by the participant: nausea, vomiting, taste changes, vaginal irritation, abdominal pain, diarrhoea. Serious adverse events are not anticipated in this low-risk trial but will be recorded if reported by participants.

Although lactic acid gel is considered safe for use in pregnancy and metronidazole is frequently prescribed for treatment of BV in pregnancy, patients will be asked to confirm that they are not pregnant as part of the screening process. Participants will also be asked to confirm their pregnancy status during their follow-up period. Any pregnancies reported during the period between randomisation and Week 2 will be followed up for outcomes.

### Data management

All trial data will be entered on a trial-specific database through the electronic Case Report Form (eCRF) with participants identified only by their unique trial number and initials. The database will be developed and maintained by the NCTU. Access to the database will be restricted and secure, and all data transactions will be logged in a full audit trail.

The NCTU will check site-completed eCRFs for compliance with the protocol, data consistency, missing data and timing. Sites will be asked for missing data or clarification of inconsistencies or discrepancies.

### Statistical considerations

#### Analysis of outcome measures

The analysis and reporting of the trial will be in accordance with Consolidated Standards of Reporting Trials (CONSORT) guidelines. A full Statistical Analysis Plan will be developed and agreed prior to database lock and unblinding of the analysing statistician. Continuous variables will be summarised in terms of the mean, standard deviation, median, lower and upper quartiles, minimum, maximum and number of observations. Categorical variables will be summarised in terms of frequency counts and percentages. Descriptive statistics of demographic and clinical measures will be used to assess balance between the randomised arms at baseline, but no formal statistical comparisons will be made.

The primary approach to between-group comparative analyses will be by the modified intention-to-treat method, i.e. including all participants who have been randomised and without imputation of missing outcome data. Sensitivity analyses will be conducted to investigate the impact of missing data and adherence to treatment.

The evaluation of the primary outcome will be performed using a mixed effects model for binary outcome that includes factors used in the minimisation. The comparison of lactic acid gel with oral metronidazole will be presented using the risk difference in the proportion of participants who reported resolution of symptoms at Week 2, along with the 95% confidence interval (CI).

Secondary outcomes will be analysed using appropriate regression models dependent on data type (binary, continuous, count, survival, etc.) and will include factors used in the minimisation and baseline value of the outcome where measured. The analyses of secondary outcomes will be considered supportive to the primary, and estimates and *p* values, where presented, should be interpreted in this light.

Presentation of quantitative tolerability data will be descriptive. Frequency counts and percentages of the proportion of participants reporting nausea, vomiting, taste disturbance, vaginal irritation, diarrhoea and abdominal pain will be presented by treatment arm.

#### Planned subgroup analyses

The primary analyses for symptom resolution will be investigated to determine whether treatment effectiveness differs according to the following subgroups:

1. Presence of concomitant STI (yes/no)
2. BV confirmed by positive microscopy (yes/no)
3. Type of centre at which participant presented (sexual health clinic versus GP/other clinics)

Between-group treatment effects will be provided for each subgroup, but interpretation of any subgroup effects will be based on the treatment subgroup interaction and 95% CI, estimated by fitting an appropriate interaction term in the regression models. Since the trial is powered to detect overall differences between the groups rather than interactions of this kind, these subgroup analyses will be regarded as exploratory.

#### Planned interim analysis

There is no planned interim analysis of treatment efficacy. However, an assessment of recruitment and adherence to treatment will be performed using data from the first 6 months of participant recruitment. This is done to determine how feasible it is that the trial is able to adequately address its primary and secondary objectives.

The Trial Steering Committee (TSC) and Data Monitoring Committee (DMC) will use the following criteria as a guide to determine whether the trial should progress:

1. Reviewing the number of participants completing their Week 2 assessment against the following targets:
  > 90%, continue the trial
  65–90%, review recruitment and retention procedures to identify underlying problems and implement strategies to address these problems, with review in 6 months
  35–65%, review recruitment and retention procedures to identify underlying problems and implement strategies to address them. Ongoing review over 6 months; terminate the trial if the recruitment trajectory does not indicate that full recruitment can occur within an acceptable recruitment period
  < 35%, terminate the trial.
2. Reviewing adherence to lactic acid gel and metronidazole against the following pre-defined targets:
  Median adherence 5–7 days per week, continue the trial
  Median adherence 3–4 days per week, review data from the qualitative interviews on adherence and tolerability to identify underlying problems and implement strategies to address them, with review in 6 months
  Median adherence < 3 days per week, terminate the trial.

#### Power calculation/sample size calculation

Assuming that 80% of participants receiving oral metronidazole achieve resolution of symptoms, 1710 participants are required for analysis to detect a 6% increase in response rate to 86% in participants receiving lactic acid gel (risk ratio 1.08) at the 5% SL (two-sided) with 90% power.

To allow for non-collection of the primary outcome from up to 10%, e.g. due to loss to follow-up, a total of 1900 participants will be recruited.

#### Health economics

The economic analysis will compare the costs associated with the current standard treatment, metronidazole, with those of the proposed alternative treatment, lactic acid gel, in the treatment of BV.

The economic evaluation will adopt the perspective of the NHS, and data on resource use and costs will be collected prospectively within the study. Resource use data will be collected prospectively via nurse/assessor/participant completed forms and will include questions on treatment use, GP or other clinic visits to estimate the costs associated with the administration of both treatments. The main resources to be monitored include: (1) additional staff time for explaining the lactic acid intervention and responding to concerns associated with treatment; (2) time and resources associated with clinical examination, additional medication and monitoring during the follow-up period as well as treating any adverse events; (3) the costs associated with treatment, for example the costs of lactic acid gel and applicators.

Information on unit costs or prices will be attached to each resource item in order that an overall cost per patient successfully treated at 2 weeks, 3 months and 6 months can be calculated, and a cost per quality-adjusted life year (QALY) at 26 weeks. The cost associated with antibiotic resistance to add as a penalty cost for using antibiotics has been estimated [22], and within this study we will explore whether a similar approach could be taken.

Outcomes will be measured in natural units according to assessment of symptoms of BV at 2 weeks, 3 months and 6 months post intervention.

#### Qualitative data analysis

Data from participant interviews will be coded thematically, with codes based on interview questions and emergent themes. Coded data will be compared between participants in the same arm of the trial, and between trial arms, and will be synthesised using a framework approach.

### Trial governance

The Trial Management Group (TMG) are responsible for day-to-day management of the trial, including review of protocol deviations entered into the eCRF. The TMG include the Chief Investigator, Trial Manager, Trial Statistician and other members of the NCTU multidisciplinary team as appropriate. The TMG are responsible for ensuring project milestones are achieved.

A TSC has been established, and their role is to provide trial oversight, monitor trial progress and conduct and advise on scientific credibility. The TSC will consider and act, when appropriate, upon the recommendations of the DMC. The role of the DMC is to monitor accruing data and make recommendations to the TSC on whether there are any ethical or safety reasons why the trial should not continue.

## Discussion

This trial uses a pragmatic design to maximise its relevance to clinicians and participants, to help ensure rapid adoption into clinical practice and to provide value for money. If effective, the use of lactic acid gel will provide an alternative treatment for women who have failed to respond to previous therapy for BV, benefitting both patients and the NHS. It will also result in decreased antibiotic use, which will maintain the balance of the gut bacteria (microbiome) for individually treated participants in addition to reducing the potential for the development of antimicrobial resistance in the community. This is an important area for clinical research, and results from the trial will help inform UK treatment guidelines for BV in addition to supporting the Department of Health’s Antimicrobial Resistance Strategy [23].

### Trial status

The protocol is version 1.0, dated 29 June 2017. Recruitment opened on 30 October 2017 and is expected to continue to 30 November 2019.

## Supplementary information

**Additional file 1.** VITA SPIRIT 2013 checklist: recommended items to address in a clinical trial protocol and related documents.

## Data Availability

Data sharing is not applicable to this article, as no datasets were generated or analysed during the current study.

## Abbreviations

BV: Bacterial vaginosis
DMC: Data Monitoring Committee
eCRF: Electronic Case Report Form
GP: General Practitioner
HIV: Human immunodeficiency virus
IMP: Investigational medicinal product
INR: International normalised ratio
ISRCTN: International Standard Randomised Controlled Trial Number
NCTU: Nottingham Clinical Trials Unit
NHS: National Health Service
NIHR HTA: National Institute for Health Research Health Technology Assessment
QALY: Quality-adjusted life year
SmPC: Summary of product characteristics
SOP: Standard operating procedure
STI: Sexually transmitted infection
TMG: Trial Management Group
TSC: Trial Steering Committee

## References

[1] Atashili J, Poole C, Ndumbe PM, Adimora AA, Smith JS (2008). Bacterial vaginosis and HIV acquisition: a meta-analysis of published studies. AIDS (London, England).

[2] Cohen CR, Lingappa JR, Baeten JM, Ngayo MO, Spiegel CA, Hong T, Donnell D, Celum C, Kapiga S, Delany S (2012). Bacterial vaginosis associated with increased risk of female-to-male HIV-1 transmission: a prospective cohort analysis among African couples. PLOS Med.

[3] Ness RB, Kip KE, Hillier SL, Soper DE, Stamm CA, Sweet RL, Rice P, Richter HE (2005). A cluster analysis of bacterial vaginosis-associated microflora and pelvic inflammatory disease. Am J Epidemiol.

[4] Leitich H, Brunbauer M, Bodner-Adler B, Kaider A, Egarter C, Husslein P (2003). Antibiotic treatment of bacterial vaginosis in pregnancy: a meta-analysis. Am J Obstet Gynecol.

[5] Bradshaw CS, Morton AN, Hocking J, Garland SM, Morris MB, Moss LM, Horvath LB, Kuzevska I, Fairley CK (2006). High recurrence rates of bacterial vaginosis over the course of 12 months after oral metronidazole therapy and factors associated with recurrence. J Infect Dis.

[6] Simoes JA, Bahamondes LG, Camargo RP, Alves VM, Zaneveld LJ, Waller DP, Schwartz J, Callahan MM, Mauck CK (2006). A pilot clinical trial comparing an acid-buffering formulation (ACIDFORM gel) with metronidazole gel for the treatment of symptomatic bacterial vaginosis. Br J Clin Pharmacol.

[7] British Association for Sexual Health and HIV (BASHH) (2012). UK National Guideline for the Management of Bacterial Vaginosis 2012.

[8] Joesoef MR, Schmid GP (1995). Bacterial vaginosis: review of treatment options and potential clinical indications for therapy. Clin Infect Dis.

[9] Schwebke JR (2009). Bacterial vaginosis: are we coming full circle?. J Infect Dis.

[10] Fiorilli A, Molteni B, Milani M (2005). Successful treatment of bacterial vaginosis with a policarbophil-carbopol acidic vaginal gel: results from a randomised double-blind, placebo-controlled trial. Eur J Obstet Gynecol Reprod Biol.

[11] Boeke AJ, Dekker JH, van Eijk JT, Kostense PJ, Bezemer PD (1993). Effect of lactic acid suppositories compared with oral metronidazole and placebo in bacterial vaginosis: a randomised clinical trial. Genitourin Med.

[12] Holley RL, Richter HE, Varner RE, Pair L, Schwebke JR (2004). A randomized, double-blind clinical trial of vaginal acidification versus placebo for the treatment of symptomatic bacterial vaginosis. Sex Transm Dis.

[13] Andersch B, Forssman L, Lincoln K, Torstensson P (1986). Treatment of bacterial vaginosis with an acid cream: a comparison between the effect of lactate-gel and metronidazole. Gynecol Obstet Investig.

[14] Fredricsson B, Englund K, Weintraub L, Olund A, Nord CE (1989). Bacterial vaginosis is not a simple ecological disorder. Gynecol Obstet Investig.

[15] Decena DC, Co JT, Manalastas RM, Palaypayon EP, Padolina CS, Sison JM, Dancel LA, Lelis MA (2006). Metronidazole with Lactacyd vaginal gel in bacterial vaginosis. J Obstet Gynaecol Res.

[16] Hay PE (1998). Therapy of bacterial vaginosis. J Antimicrob Chemother.

[17] Larsson PG (1992). Treatment of bacterial vaginosis. Int J STD AIDS.

[18] Lugo-Miro VI, Green M, Mazur L (1992). Comparison of different metronidazole therapeutic regimens for bacterial vaginosis. A meta-analysis. JAMA.

[19] Hillier SL, Lipinski C, Briselden AM, Eschenbach DA (1993). Efficacy of intravaginal 0.75% metronidazole gel for the treatment of bacterial vaginosis. Obstet Gynecol.

[20] Haggerty CL, Ness RB, Amortegui A, Hendrix SL, Hillier SL, Holley RL, Peipert J, Randall H, Sondheimer SJ, Soper DE (2003). Endometritis does not predict reproductive morbidity after pelvic inflammatory disease. Am J Obstet Gynecol.

[21] Brazier J, Roberts J, Deverill M (2002). The estimation of a preference-based measure of health from the SF-36. J Health Econ.

[22] Oppong R, Smith RD, Little P, Verheij T, Butler CC, Goossens H, Coenen S, Moore M, Coast J (2016). Cost effectiveness of amoxicillin for lower respiratory tract infections in primary care: an economic evaluation accounting for the cost of antimicrobial resistance. Br J Gen Pract.

[23] Department of Health (2013). UK Five Year Antimicrobial Resistance Strategy 2013 to 2018.

